# Draft genome sequences of *Bacillus licheniformis* strains MEZBL63 and MEZBL64 harboring the lichenysin toxin operon isolated from livestock in South Africa

**DOI:** 10.1128/mra.00967-23

**Published:** 2024-02-07

**Authors:** Mohamed E. El Zowalaty, Linda Falgenhauer, Hossam M. Ashour, Oliver T. Zishiri, Stephen Forsythe, Yosra A. Helmy

**Affiliations:** 1 Veterinary Medicine and Food Security Research Group, Medical Laboratory Sciences Program, Faculty of Health Sciences, Abu Dhabi Women's Campus, Higher Colleges of Technology, Abu Dhabi, United Arab Emirates; 2 Institute of Hygiene and Environmental Medicine, German Center for Infection Research, Site Giessen-Marburg-Langen and Hessian University Competence Center for Hospital Hygiene (HuKKH), Justus Liebig University Giessen, Giessen, Germany; 3 Department of Integrative Biology, College of Arts and Sciences, University of South Florida, St. Petersburg, Florida, USA; 4 Discipline of Genetics, School of Life Sciences, College of Agriculture, Engineering and Science, University of KwaZulu-Natal, Durban, South Africa; 5 Foodmicrobe.com Ltd, Adams Hill, Keyworth, Nottingham, United Kingdom; 6 Department of Veterinary Science, Martin-Gatton College of Agriculture, Food, and Environment, University of Kentucky, Lexington, Kentucky, USA; University of Maryland School of Medicine, Baltimore, Maryland, USA

**Keywords:** *Bacillus licheniformis*, lichenysin, toxin, genome, sequence, livestock

## Abstract

Here, we report the draft genome sequences of two *Bacillus licheniformis* strains harboring the lichenysin operon that were isolated from healthy goat and horse in South Africa. The genomes were sequenced using Illumina MiSeq and had a length of 4,152,826 and 4,110,075 bp, respectively, with a G + C content of 46%.

## ANNOUNCEMENT

*Bacillus licheniformis* is a ubiquitous, endospore-forming, Gram-positive saprophytic bacterium that belongs to the *Bacillus subtilis* group (1). It is an opportunistic pathogen in compromised hosts and it is characterized by high temperature and stress resistance (2). It causes a wide range of infrequent infections including gastroenteritis, bacteremia, peritonitis, and ophthalmitis in immunocompromised humans (3–6). It can cause toxemia, mastitis, and abortion in bovine (7, 8) and is implicated in foodborne poisoning and outbreaks (9–14). Lichenysin toxin-producing strains were isolated from raw milk and infant food products (8–10). Additionally, *B. licheniformis* has biotechnological applications (15), and it was used as a potential probiotic and antibiotics alternative to prevent pathogenic infection in broilers (16–18).

Here, we report draft genome sequences of *B. licheniformis* strains MEZBL63 and MEZBL64 isolated from fecal samples collected from a horse and a goat, respectively, in Flagstaff, OR Tambo District, Eastern Cape, South Africa in May 2018. The samples were collected as part of *One Health* research project investigating zoonotic diseases and antimicrobial resistance. The samples were collected in 10 mL of 0.1% buffered peptone water and were incubated for 24 h as reported previously (19). The samples were isolated using sheep blood agar for 18–24 h at 37°C in the presence of 5% CO_2_. A single CFU was cultured in tryptic soy broth to an inoculum density of a 0.5-McFarland turbidity standard, and DNA was extracted from the cultures using DNeasy Blood & Tissue kit (Qiagen, Maryland, USA) as previously reported (19, 20). Sequencing libraries were prepared using Nextera XT Library Preparation Kit (Illumina Inc., California, USA). Sequencing was performed using Illumina MiSeq instrument (Illumina Inc., California, USA) and MiSeq Reagent Kit v2 (500 cycles). Sequencing yielded an average coverage of 29× and an average read length of 208 nt (Table 1).

**TABLE 1:** Basic characteristics of the whole-genome sequencing, assemblies, and annotation of strains MEZBL63 and MEZBL64

Parameter	MEZBL63	MEZBL64
No. of raw reads	262,351	282,025
Average read length (nt)	220	219
Average coverage (×)	27.8	30.1
No. of contigs >200 bp	183	183
N_50_ (bp)	36,931	36,462
Genome size (bp)	4,152,826	4,110,075
G + C content (%)	46	46
No. of genes (total)	4,350	4,275
No. of CDSs (with protein)	4,204	4,118
No. of genes (RNA)	73	78
No. of rRNAs (5S, 16S, 23S)	1, 1, 1	1, 1, 1
Complete	1, 1, 1	1, 1, 1
Partial	0, 0, 0	0, 0, 0
No. of tRNAs	67	70
No. of ncRNAs	3	5
No. of pseudogenes (total)	73	79
Multilocus sequence type	3	3

Trimming (Trimmomatic v.0.36) (21), quality control (fastqc v.0.11.) (22), and assembly of raw data (SPAdes version 3.12.0) (23) were performed within the framework of ASA^3^P pipeline (v.1.4.0) (24). Contigs smaller than 200 nucleotides were discarded. Genome annotations were performed using NCBI Prokaryotic Genome Annotation Pipeline ( v.5.3) (25). The species were determined using TYGS pipeline (26). Sequencing, assembly, and annotation data of MEZBL63 and MEZBL64 are shown in Table 1. Assembly yielded a total length of 4,152,826 bp for MEZBL63 and 4,110,075 bp for MEZBL64, respectively. The GC content detected was 46%. An *N*_50_ value of 36,931 bp (MEZBL63) and 36,462 bp (MEZBL64) was achieved.

Multilocus sequence type determination was performed using PubMLST database and the previously established genotyping scheme for *B. licheniformis* (27). Both MEZBL63 and MEZBL64 strains belong to sequence type 3.

Analysis of the genomes using ResFinder (v.4.1) (28) and PlasmidFinder (v2.1) (29) revealed that MEZBL63 and MEZBL64 harbored neither antibiotic resistance genes nor plasmids. Virulence genes were analyzed using VFDB database implemented in ASA^3^P (24), and no virulence genes were detected.

BLAST analysis using blast stand-alone version 2.10.0+ was performed in prfectblast v.2.0 (30) to determine the presence/absence of lichenysin operon (reference sequence: accession number GU949560.1) in both genomes. This analysis revealed that MEZBL63 and MEZBL64 harbored lichenysin operon (licABC/TE) albeit with several differences in the amino acid sequence, thus supporting the theory that they are two different isolates.

## Data Availability

The whole-genome sequences were deposited at DDBJ/ENA/GenBank under the BioProject number PRJNA716986 (BioSample accession numbers SAMN23419540 and SAMN23419541, GenBank accession numbers JAJNNM000000000 and JAJNNL000000000 for MEZBL63 and MEZBL64, respectively. The versions described in this paper are the first versions. The sequences have been submitted to the Sequence Read Archive (SRA) under the accession numbers SRR17485928 and SRR17485927.

## References

[1] Fritze D. 2002. Bacillus identification – traditional approaches, p 100–122. In Berkeley R, Heyndrickx M, Logan N, De P (ed), Applications and Systematics of Bacillus and relatives. Wiley-Blackwell, UK.

[2] Wecke T, Veith B, Ehrenreich A, Mascher T. 2006. Cell envelope stress response in Bacillus licheniformis: integrating comparative genomics, transcriptional profiling, and regulon mining to decipher a complex regulatory network. J Bacteriol 188:7500–7511. doi:10.1128/JB.01110-06 16936031 PMC1636251

[3] Hoi LT, Voigt B, Jürgen B, Ehrenreich A, Gottschalk G, Evers S, Feesche J, Maurer K-H, Hecker M, Schweder T. 2006. The phosphate-starvation response of Bacillus licheniformis. Proteomics 6:3582–3601. doi:10.1002/pmic.200500842 16705752

[4] Tabbara KF, Tarabay N. 1979. Bacillus licheniformis corneal ulcer. Am J Ophthalmol 87:717–719. doi:10.1016/0002-9394(79)90311-8 443345

[5] Thurn JR, Goodman JL. 1988. Post-traumatic ophthalmitis due to Bacillus licheniformis. Am J Med 85:708–710. doi:10.1016/s0002-9343(88)80246-8 3263801

[6] Blue SR, Singh VR, Saubolle MA. 1995. Bacillus licheniformis bacteremia: Five cases associated with indwelling central venous catheters. Clin Infect Dis 20:629–633. doi:10.1093/clinids/20.3.629 7756487

[7] Johnson CT, Lupson GR, Lawrence KE. 1994. The bovine placentome in bacterial and mycotic abortions. Vet Rec 134:263–266. doi:10.1136/vr.134.11.263 8197693

[8] Nieminen T, Rintaluoma N, Andersson M, Taimisto AM, Ali-Vehmas T, Seppälä A, Priha O, Salkinoja-Salonen M. 2007. Toxinogenic Bacillus pumilus and Bacillus licheniformis from mastitic milk. Vet Microbiol 124:329–339. doi:10.1016/j.vetmic.2007.05.015 17611049

[9] Banykó J, Vyletelová M. 2009. Determining the source of Bacillus cereus and Bacillus licheniformis isolated from raw milk, pasteurized milk and yoghurt. Lett Appl Microbiol 48:318–323. doi:10.1111/j.1472-765X.2008.02526.x 19187503

[10] Salkinoja-Salonen MS, Vuorio R, Andersson MA, Kämpfer P, Andersson MC, Honkanen-Buzalski T, Scoging AC. 1999. Toxigenic strains of Bacillus licheniformis related to food poisoning. Appl Environ Microbiol 65:4637–4645. doi:10.1128/AEM.65.10.4637-4645.1999 10508100 PMC91618

[11] Logan NA. 2012. Bacillus and relatives in foodborne illness. J Appl Microbiol 112:417–429. doi:10.1111/j.1365-2672.2011.05204.x 22121830

[12] Lund BM. 1990. Foodborne diseases due to Bacillus and Clostridium species. Lancet 336:982–986. doi:10.1016/0140-6736(90)92431-g 1977014

[13] Rosenkvist H, Hansen Å. 1995. Contamination profiles and characterization of Bacillus species in wheat bread and raw materials for bread production. Int J Food Microbiol 26:353–363. doi:10.1016/0168-1605(94)00147-x 7488530

[14] Tatzel R, Ludwig W, Schleifer KH, Wallnöfer PR. 1994. Identification of Bacillus strains isolated from milk and cream with classical and nucleic acid hybridization methods. J Dairy Res 61:529–535. doi:10.1017/s0022029900028454 7829756

[15] Muras A, Romero M, Mayer C, Otero A. 2021. Biotechnological applications of Bacillus licheniformis. Crit Rev Biotechnol 41:609–627. doi:10.1080/07388551.2021.1873239 33593221

[16] Chen YC, Yu YH. 2022. Bacillus licheniformis-fermented products and enramycin differentially modulate microbiota and antibiotic resistome in the cecal digesta of broilers. Poult Sci 101:102010. doi:10.1016/j.psj.2022.102010 35841645 PMC9293667

[17] Gong L, Wang B, Mei X, Xu H, Qin Y, Li W, Zhou Y. 2018. Effects of three probiotic Bacillus on growth performance, digestive enzyme activities, antioxidative capacity, serum immunity, and biochemical parameters in broilers. Anim Sci J 89:1561–1571. doi:10.1111/asj.13089 30198073

[18] Abudabos AM, Aljumaah MR, Alkhulaifi MM, Alabdullatif A, Suliman GM, R Al Sulaiman A. 2020. Comparative effects of Bacillus subtilis and Bacillus licheniformis on live performance, blood metabolites and intestinal features in broiler inoculated with Salmonella infection during the finisher phase. Microb Pathog 139:103870. doi:10.1016/j.micpath.2019.103870 31734387

[19] El Zowalaty ME, Moura A, Lecuit M, Zishiri OT. 2022. Draft genome sequence of Listeria innocua strain MEZLIS29, isolated from a cow in South Africa. Microbiol Resour Announc 11:e0112221. doi:10.1128/mra.01122-21 35225692 PMC8928759

[20] El Zowalaty ME, Falgenhauer L, Forsythe S, Helmy YA. 2023. Draft genome sequences of rare Lelliottia nimipressuralis strain MEZLN61 and two Enterobacter kobei strains MEZEK193 and MEZEK194 carrying mobile colistin resistance gene mcr-9 isolated from wastewater in South Africa. J Glob Antimicrob Resist 33:231–237. doi:10.1016/j.jgar.2023.03.007 36948496

[21] Bolger AM, Lohse M, Usadel B. 2014. Trimmomatic: a flexible trimmer for illumina sequence data. Bioinformatics 30:2114–2120. doi:10.1093/bioinformatics/btu170 24695404 PMC4103590

[22] Andrews S. 2023. Babraham Bioinformatics. FastQC: a quality control tool for high throughput sequence data. Babraham Institute, Cambridge, United Kingdom. Available from: https://www.bioinformatics.babraham.ac.uk/projects/fastqc

[23] Bankevich A, Nurk S, Antipov D, Gurevich AA, Dvorkin M, Kulikov AS, Lesin VM, Nikolenko SI, Pham S, Prjibelski AD, Pyshkin AV, Sirotkin AV, Vyahhi N, Tesler G, Alekseyev MA, Pevzner PA. 2012. SPAdes: a new genome assembly algorithm and its applications to single-cell sequencing. J Comput Biol 19:455–477. doi:10.1089/cmb.2012.0021 22506599 PMC3342519

[24] Schwengers O, Hoek A, Fritzenwanker M, Falgenhauer L, Hain T, Chakraborty T, Goesmann A. 2020. ASA3P: an automatic and scalable pipeline for the assembly, annotation and higher-level analysis of closely related bacterial isolates. PLoS Comput Biol 16:e1007134. doi:10.1371/journal.pcbi.1007134 32134915 PMC7077848

[25] Tatusova T, DiCuccio M, Badretdin A, Chetvernin V, Nawrocki EP, Zaslavsky L, Lomsadze A, Pruitt KD, Borodovsky M, Ostell J. 2016. NCBI prokaryotic genome annotation pipeline. Nucleic Acids Res 44:6614–6624. doi:10.1093/nar/gkw569 27342282 PMC5001611

[26] Meier-Kolthoff JP, Carbasse JS, Peinado-Olarte RL, Göker M. 2022. TYGS and LPSN: a database tandem for fast and reliable genome-based classification and nomenclature of prokaryotes. Nucleic Acids Res 50:D801–D807. doi:10.1093/nar/gkab902 34634793 PMC8728197

[27] Madslien EH, Olsen JS, Granum PE, Blatny JM. 2012. Genotyping of B. licheniformis based on a novel multi-locus sequence typing (MLST) scheme. BMC Microbiol 12:230. doi:10.1186/1471-2180-12-230 23051848 PMC3492095

[28] Bortolaia V, Kaas RS, Ruppe E, Roberts MC, Schwarz S, Cattoir V, Philippon A, Allesoe RL, Rebelo AR, Florensa AF, et al.. 2020. ResFinder 4.0 for predictions of phenotypes from genotypes. J Antimicrob Chemother 75:3491–3500. doi:10.1093/jac/dkaa345 32780112 PMC7662176

[29] Carattoli A, Hasman H. 2020. Plasmidfinder and in silico pMLST: identification and typing of plasmid replicons in whole-genome sequencing (WGS). Methods Mol Biol 2075:285–294. doi:10.1007/978-1-4939-9877-7_20 31584170

[30] Santiago-Sotelo P, Ramirez-Prado JH. 2012. prfectBLAST: a platform-independent portable front end for the command terminal BLAST+ stand-alone suite. Biotechniques 53:299–300. doi:10.2144/000113953 23148880

